# A mixed‐methods systematic review of nurse‐led interventions for people with multimorbidity

**DOI:** 10.1111/jan.15427

**Published:** 2022-09-05

**Authors:** Chris McParland, Bridget Johnston, Mark Cooper

**Affiliations:** ^1^ School of Medicine Dentistry and Nursing University of Glasgow Glasgow Scotland; ^2^ NHS Greater Glasgow and Clyde Glasgow Scotland

**Keywords:** comorbidity, literature review, mixed‐methods, multimorbidity, nurse‐led care, nurses, nurse's practice patterns, nursing, systematic review

## Abstract

**Aims:**

To identify types of nurse‐led interventions for multimorbidity and which outcomes are positively affected by them.

**Design:**

Mixed‐methods systematic review following the Joanna Briggs Institute (JBI) methods for convergent‐integrated reviews. PROSPERO ID: CRD42020197956.

**Data Sources:**

Cochrane CENTRAL, CINAHL, Embase and MEDLINE were searched in October 2020. Grey literature sources included OpenGrey, the Journal of Multimorbidity and Comorbidity and reference mining.

**Review Methods:**

English‐language reports of nurse‐led interventions for people with multimorbidity were included based on author consensus. Two reviewers performed independent quality appraisal using JBI tools. Data were extracted and synthesized using a pre‐existing taxonomy of interventions and core outcome set.

**Results:**

Twenty studies were included, with a median summary quality score of 77.5%. Interventions were mostly case‐management or transitional care interventions, with nurses in advanced practice, support to self‐manage conditions, and an emphasis on continuity of care featuring frequently. Patient‐centred outcomes such as quality of healthcare and health‐related quality of life were mostly improved, with mixed effects on healthcare utilization, costs, mortality and other outcomes.

**Conclusion:**

Interventions such as case management are agreeable to patients and transitional care interventions may have a small positive impact on healthcare utilization. Interventions include long‐term patient management or short‐term interventions targeted at high‐risk junctures. These interventions feature nurses in advanced practice developing care plans in partnership with patients, to simplify and improve the quality of care both in the long and short‐term.

**Impact:**

This is the first mixed‐methods review which includes all types of nurse‐led interventions for multimorbidity and does not focus on specific comorbidities or elderly/frail populations. Using adapted consensus‐developed frameworks for interventions and outcomes, we have identified the common features of interventions and their overall typology. We suggest these interventions are of value to patients and healthcare systems but require localization and granular evaluation of their components to maximize potential benefits.

## 1 INTRODUCTION

People are living longer now than ever before and spending an increasing number of years in worsening health. The proportion of people living in the community with multimorbidity (two or more chronic conditions) is estimated to be around 33% (Nguyen et al., 2019), growing significantly with advanced age and increasing deprivation (Head et al., 2021). The cumulative burden of disease alongside socioeconomic drivers of poor health mean that those with multimorbidity are at increased risk of mortality, disability, functional decline, increased healthcare use and reduced quality of life (Xu et al., 2017). Isolation compounds this risk, as older people with multimorbidity who live alone are more likely to require emergency care than those who live with others (Barrenetxea et al., 2021). The structure of healthcare services also places people with multimorbidity at risk of care fragmentation and treatment burden (Morris et al., 2021).

Nurse‐led care delivered by experienced nurses is comparable in quality to physician‐led primary care (Laurant et al., 2018), but it should not simply be considered a replacement for doctor‐led care. Nursing care is traditionally holistic and if provided in the context of a supportive and adequately resourced organization, builds on the foundation of a therapeutic relationship where patient's goals and wishes are central to their care (Bridges et al., 2013). Nursing interventions are ideally suited to supporting people with multimorbidity, for whom priorities may change with time and are not necessarily tied to specific conditions. To do this effectively, nurses require a robust evidence base to inform their practice (O'Connor et al., 2018).

## 2 BACKGROUND

### 2.1 Multimorbidity

Multimorbidity refers to the coexistence of two or more chronic conditions (van den Akker et al., 1996). Comorbidity also refers to concurrent chronic conditions, yet it differs from multimorbidity by focussing on a specific index condition to which others are considered comorbid (Feinstein, 1970).To adopt a multimorbidity framework in research or clinical practice requires one to accept that no condition holds precedence, and that clinical decisions are taken within the overall context of the person (Boyd & Fortin, 2010).

This distinction matters in the way interventions are developed and evaluated. Interventions for index conditions and comorbidities can be targeted and use disease‐specific outcomes but may not be generalizable to people with multimorbidity. Multimorbidity interventions will be more generic in their design, and the selection of outcomes to measure their effectiveness is more difficult (Harrison et al., 2021).

We must also consider what constitutes a chronic condition. Diagnosed diseases such as asthma or diabetes meet this requirement. But in the context of the person, symptoms (such as breathlessness or pain) or risk factors (such as obesity or poverty) are also important (Willadsen et al., 2016). The number of chronic conditions which are considered is also a concern (Fortin et al., 2012), as identifying multimorbidity from a small number of conditions may limit the generalizability of findings.

The strongest evidence to support interventions for concurrent conditions comes from studies which focus on specific disease clusters, or improving outcomes of common comorbidities, such as depression (Smith et al., 2021). Most multimorbidity interventions are evaluated using disease‐specific outcomes (Xu et al., 2017), theoretically limiting the generalizability of these effects to groups with different combinations of disease. Approaches which are patient‐oriented and support self‐management are increasingly viewed as essential (Poitras et al., 2018; Smith et al., 2021).

### 2.2 Nurse‐led care

The concept of person‐centred (or person‐oriented) care underpins much of contemporary health policy but is particularly associated with nursing, providing a theoretical framework on which nursing interventions can be planned, implemented and evaluated. Key processes in person‐centred nursing include engaging with patients, shared decision‐making, creating a sympathetic presence, providing for physical needs and working with the patient's beliefs and values. Evaluating the effectiveness of such interventions should incorporate patient satisfaction, the level of involvement they have with care, their feelings of well‐being, and the presence of a therapeutic culture (McCormack & McCance, 2006).

Community‐based nurse‐led interventions cover a diverse range of areas including walk‐in clinics, primary care clinics, smoking cessation, women's health and healthcare for homeless persons. These types of interventions have been shown to improve access to healthcare, symptom management, and a range of disease‐specific measures (Randall et al., 2017). Conversely, there is strong evidence to suggest that peri‐discharge interventions of varying complexity are not effective in reducing hospital reattendance when compared with usual care (Wong et al., 2021). We cannot, however, generalize these findings to people with multimorbidity.

### 2.3 Definitions

Based on the work of Boyd and Fortin (2010), Le Reste et al. (2013) and Willadsen et al. (2016), we define multimorbidity as the coexistence of two or more chronic conditions, where no condition holds precedence. Conditions are not restricted to diagnosed chronic diseases and can include biopsychosocial and somatic risk factors.

Nurse‐led interventions were required to satisfy at least one of the following requirements: (1) the service is led by a senior (possibly consultant) nurse, (2) nurses manage and are accountable for a case‐load of patients or (3) nurses practice with a discernible degree of autonomy compared with medically led care.

### 2.4 Rationale for review

Previous reviews established that interventions which are person‐oriented and incorporate self‐management have the potential to improve outcomes in persons with multimorbidity (Crowe et al., 2016; Poitras et al., 2018; Smith et al., 2021). However, the evidence‐base for certain types of nursing interventions for this group has not provided robust evidence of their impact on a wide range of outcomes (Lupari et al., 2011). Given the important role person‐centred care plays in nursing practice (McCormack & McCance, 2006), we believe a review which seeks to categorize and evaluate nurse‐led interventions for people with multimorbidity is warranted.

## 3 THE REVIEW

### 3.1 Aims

This review aims to identify what types of nurse‐led interventions are effective in improving outcomes for people with multimorbidity.

#### 3.1.1 Specific objectives

1. To identify and categorize the different types of nurse‐led interventions for people with multimorbidity, and
2. To identify which outcomes are improved by nurse‐led interventions for people with multimorbidity.

### 3.2 Design

This review follows the Joanna Briggs Institute (JBI) guidance for mixed‐methods systematic reviews using a convergent‐integrated approach (Lizarondo et al., 2020). This involves simultaneous analysis of qualitative and quantitative findings. Evidence grading is not recommended due to heterogeneity of sources. We have instead sought to classify whether different interventions have an effect which is best described as ‘mostly improved’, ‘mostly unaffected’, or ‘mixed’. The review was registered on PROSPERO in October 2020 (ID: CRD42020197956), and is reported following the Preferred Reporting Items for Systematic Reviews and Meta‐analyses (PRISMA) 2021 guidance (Page et al., 2021).

### 3.3 Search methods

#### 3.3.1 Databases

Cochrane CENTRAL, CINAHL, Embase and MEDLINE were searched from inception using the terms in Table 1, which were based on a comprehensive list of synonyms from a recent systematic review (Makovski et al., 2019). All searches were conducted in October 2020. File S1 details search strategies.

**TABLE 1:** Search terms employed

Multimorbidity terms	Nurse‐led care terms		
Multimorbidity	Nurse led		
Multi‐morbidity	Nurse‐led		
Multi morbidity	Nurse managed		
Multimorbidities	Nurse‐managed		
Multi‐morbidities	Nurse based intervention		
Multi morbidities	Nurse‐based intervention		
Multimorbid	Primary nurse		
Multi‐morbid	Primary nurses		
Multi morbid	Primary nursing		
Comorbidity	Nurse practitioner		
Co‐morbidity	Nurse practitioners		
Co morbidity	Practitioner nurse		
Comorbidities	Practitioner nurses		
Co‐morbidities	Advanced practice nurse		
Co morbidities	Advanced practice nurses		
Comorbid	Advanced practice nursing		
Co‐morbid	Nurse specialist		
Co morbid	Nurse specialists		
Multiple chronic conditions	Specialist nurse		
Multiple chronic illnesses	Specialist nurses		
Multiple chronic diseases	Specialist nursing		
Multiple conditions	Nurse clinician		
Multiple illnesses	Nurse clinicians		
Multiple diseases	Nurse consultant		
Multiple diagnoses	Nurse consultants		
Morbidity pattern	Consultant nurse		
Morbidity patterns	Consultant nurses		
Polymorbidity	Case manager	**AND**	(nurse OR nurses OR nursing)
Poly‐morbidity	Case‐manager	**AND**	
Poly morbidity	Case management	**AND**	
Polymorbidities	Case‐management	**AND**	
Poly‐morbidities			
Poly morbidities			
Polypathology			
Poly‐pathology			
Poly pathology			
Polypathologies			
Poly‐pathologies			
Poly pathologies			
Pluripathology			
Pluri‐pathology			
Pluri pathology			
Multipathology			
Multi‐pathology			
Multi pathology			
Multipathologies			
Multi‐pathologies			
Multi pathologies			
Multiple pathologies			
Disease cluster			
Disease clusters			

#### 3.3.2 Additional sources

OpenGrey and the Journal of Multimorbidity and Comorbidity were searched in February 2021 (see File S1). The reference lists of articles included in the review, systematic reviews identified during scoping (Crowe et al., 2016; Poitras et al., 2018; Smith et al., 2021) and screening (Deschodt et al., 2020; Gordon et al., 2019; Latour et al., 2007; Lupari et al., 2011; Massimi et al., 2017; Morilla‐Herrera et al., 2016) were also searched.

#### 3.3.3 Inclusion/exclusion criteria

Peer‐reviewed research using qualitative, quantitative or mixed‐methods to evaluate nurse‐led interventions for people with multimorbidity were eligible. We applied the definitions of nurse‐led care and multimorbidity as outlined earlier. Only English‐language sources were included. Articles which dealt with specific clusters of conditions, only mental‐health conditions or those which assumed multimorbidity purely on the basis of advanced age or frailty were excluded.

#### 3.3.4 Screening for eligibility

The first author applied inclusion/exclusion criteria to titles/abstracts and borderline decisions were agreed among the remaining authors. The first author read all full‐text articles, with the role of second reviewer being shared between the second/third authors. Decisions were agreed between the group. Endnote (The EndNote Team, 2013) and Rayyan (Ouzzani et al., 2016) were used to manage and screen citations.

### 3.4 Search outcome

**FIGURE 1:**
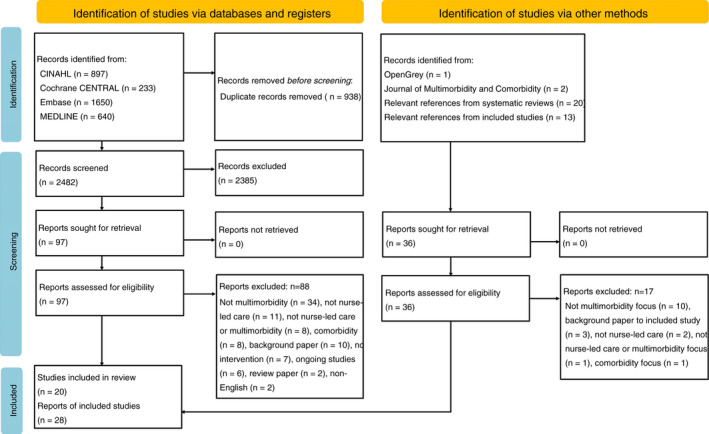
PRISMA flow diagram

The search outcome is detailed in Figure 1. Three thousand four hundred and twenty citations were retrieved, deduplicated and screened. Ninety‐seven full‐text articles were read and 88 were excluded, mostly for not being related to multimorbidity (*n* = 34), for not detailing a nurse‐led intervention (*n* = 11), or a combination of both (*n* = 8). Thirty‐six articles were retrieved through other means as detailed in Figure 1, of which 19 were eligible for inclusion. The final review includes 28 reports detailing 20 studies.

### 3.5 Quality appraisal

Studies were independently appraised by two authors using JBI tools, and discrepancies were resolved through discussion between all three authors. A summary quality score (the percentage of checklist outcomes satisfied) was calculated for each study. Studies were not excluded based on quality. Full details are given in File S2.

### 3.6 Data extraction and abstraction

Data were extracted using JBI tools. Citation, methodology, participant details, intervention details, setting, outcomes affected, and authors' conclusions were extracted. Intervention details were extracted qualitatively and background papers were consulted as necessary. All quantitative findings related to the review questions were extracted. Meta‐analysis was not attempted, primarily due to heterogeneity in intervention types, but also in study designs, populations, multimorbidity definitions and outcome measures. Qualitative data were extracted at the level of themes, subthemes or other distinct concepts outlined in the results sections of included papers.

### 3.7 Synthesis

A convergent‐integrated approach requires that quantitative data be synthesized narratively so it can be integrated with the qualitative findings. Data extraction forms were imported into NVIVO 12 (QSR International Pty Ltd, 2018), and synthesis was theoretically guided by the use of existing frameworks alongside inductive coding.

#### 3.7.1 Interventions

The Cochrane Effective Practice and Organization of Care (EPOC) taxonomy of interventions (EPOC, 2015) provided a framework to describe interventions. Studies were indexed at two levels: components and interventions. Components were defined as *any described part of a complex intervention which is being delivered as an alternative to standard care*. The intervention type was defined as *the aspect of an intervention which most accurately summarizes the entire intervention*. For example if an intervention comprised the introduction of a nurse case‐manager to coordinate care, and that case manager was able to provide home visits and transitional care, we would classify the intervention type as *case‐management*, and the components as *(i) nurse home visits* and *(ii) transitional care*. To ensure that novel aspects of interventions were captured, inductive coding was also conducted.

#### 3.7.2 Outcomes

The Core Outcome Set for Multimorbidity Research (COSmm; Smith et al., 2018) details outcomes important to multimorbidity research. Extracted data were indexed under the 17 outcomes in the COSmm, and classified as positive, negative or unaffected. For quantitative data, *p* < .05 was considered significant and any appropriate effect size measurement was considered. Qualitative evidence deemed credible or unequivocal during extraction was also indexed under relevant outcomes. To avoid exclusion of relevant findings which did not fit within this framework, Inductive coding of outcomes was also undertaken by the first author.

## 4 RESULTS

### 4.1 Characteristics of included studies

Table 2 outlines included studies. Most employed quantitative or mixed‐methods (*n* = 17), half were from the United States (*n* = 8) and Canada (*n* = 2), followed by the United Kingdom (*n* = 3), Spain (*n* = 2) and Sweden (*n* = 2). Hong Kong, Israel and Portugal each had one study. Quantitative studies were mostly quasi‐experimental (*n* = 10), and all cohort studies (*n* = 4) were retrospective. Only three randomized controlled trials (RCTs) were found, and these included cluster and pragmatic designs. Qualitative studies (*n* = 4) and the qualitative component of mixed‐methods studies (*n* = 2) were mostly descriptive or exploratory.

**TABLE 2:** Summary of included studies

Study, location and report(s) cited^a^	Design	Participants	Summary quality score^b^	Intervention	Summary of key findings [outcome measure]^c^
Boyd et al., 2008 USA (Boyd et al., 2008; Sylvia et al., 2008)	Controlled (quasi‐experimental) clinical trial (pilot)	150 (75 intervention, 75 control) Age > 65, multimorbid population identified using predictive modelling	67%	Guided Care Primary care nurse case‐management intervention. Pilot tested 6 of 8 components	**Quality of healthcare—mixed** No significant improvement in any domain of patient satisfaction [PACIC] in intention to treat and per‐protocol analyses. One domain (communication) significant improvement in regression model **Costs—mixed** Possible lower healthcare expenditure for Guided Care patients at lower risk of healthcare utilization
Boult et al., 2011 USA (Boult et al., 2008, 2011; Boyd et al., 2010; Leff et al., 2009)	Cluster randomized controlled trial	904 (485 intervention, 419 control) Age > 65, multimorbid population identified using predictive modelling	77%	Guided Care Primary care nurse case‐management intervention	**Quality of healthcare—mostly improved** Small but significant improvement in overall patient satisfaction [PACIC] Some aspects of physician satisfaction improved but mostly unaffected [PCAT] 6/7 GC nurses moderately/very satisfied with role **Healthcare use—mostly unaffected** GC did not reduce emergency, secondary care, primary care or skilled nursing facility usage. Reduced home healthcare episodes. **Costs—mostly unaffected** No significant reduction in costs for GC patients
Chow & Wong, 2014 Hong Kong (Chow & Wong, 2014)	Randomized controlled trial (3 arms)	281 (98 control, 87 home visit arm, 96 phone arm) Age > 65, 2 or more conditions and admitting diagnosis of chronic respiratory disease, cardiac disease, T2DM or renal disease	85%	Transitional care intervention for hospital discharged adults. Delivered by advanced practice nurses and nursing students. One arm received home visits and telephone calls, one arm received telephone calls only	**Healthcare use—mixed** Readmission rates reduced at 84 days post‐discharge in both intervention arms vs. control, significant in phone arm vs. control. No significant improvement in readmission at 28 days. **Health‐related quality of life—mixed** Physical component of [SF‐36] higher in intervention arms at baseline, 28 and 84 days. No significant difference in mental component [SF‐36] **Self‐efficacy—mostly improved** Both intervention arms had higher self‐efficacy across all three timepoints compared with control [Short‐form Chronic Disease Self‐efficacy scale] **Self‐rated health—mostly improved** Evidence of improvement in self‐rated health in intervention groups at 28 and 84 days [Likert‐type scale]
Dorr et al., 2008 USA (Dorr et al., 2008)	Controlled (quasi‐experimental) clinical trial	3432 (1144 intervention, 2288 control) Age ≥ 65, complexity including multimorbidity referring criteria	89%	Intermountain nurse case management intervention (primary care based)	**Mortality—mixed** Deaths were lower in intervention group at 1 year, no difference by 2 years. **Healthcare use—mostly unaffected** No significant improvement in hospitalization and ED use were detected. Improvements were noted across outcomes in a diabetes subgroup
Garcia‐Fernandez et al., 2014 Spain (García‐Fernández et al., 2014)	Retrospective analytical cohort study	255 (62 intervention, 193 control) Age ≥ 18, requirement that participants were complex including >2 diseases	55%	Hospital‐based nurse case manager	**Mortality—mostly unaffected** No difference between groups in mortality at 90 days **Healthcare use—mostly unaffected** Non‐significant difference between groups in readmission or resource utilization at 90 days **Activities of daily living—mostly improved** Intervention group had stable [Barthel index] score while control group declined **Quality of healthcare—mostly improved** Satisfaction with care and care continuity was higher in the case‐managed group at 90 days **Caregiver support—mixed** [Caregiver Burden Index] remained stable in intervention group while control declined. No differences detected in [Caregiver Preparation Index]
Hanson et al., 2018 USA (Hanson et al., 2018; Takahashi et al., 2016)	Retrospective analytical cohort study	730 (365 intervention and 365 propensity‐matched cohort) Age ≥ 60, multimorbid population identified using predictive modelling	100%	Mayo Clinic Care Transitions programme. Advanced practice nurse transitional care intervention	**Mortality—mixed** Significant reduction in mortality for intervention group at 30 days, not sustained at 90 or 180 days **Healthcare use—mixed** Significant reduction in readmissions and readmission/ED visits for the intervention at 30 days, not sustained at 90 or 180 days. No differences in bed days, ICU days, ED visits at any timepoint. **Costs—mixed** No significant reductions in costs for intervention group at 30 or 90 days. Further analysis revealed significant reduction at 30 days for the 80th percentile risk group
Hjelm et al., 2015 Sweden (Gustafsson et al., 2013; Hjelm et al., 2015)	Qualitative (focussed ethnography)	9 case managers (8 were nurses) 13 older (>75) people with multimorbidity (≥3 conditions and ≥3 hospital admissions in last year)	90%	Blekinge case management intervention Operates from outside healthcare system, CMs are non‐clinical	**Quality of healthcare—mostly improved** Several illustrations highlighting how patients appreciated the CMs ability to develop therapeutic relationships with those in their care **Trust and advocacy—mostly improved** Several illustration highlighting that the CMs were trusted by patients and appreciated for their role in advocating for the patient
Hummel et al., 2017 USA (Hummel et al., 2017)	Retrospective analytical cohort study	82 (19 intervention, 63 control) Veterans Health Association patients, multimorbid population identified using predictive modelling	55%	Intensive Management Patient Aligned Care Team (imPACT) Nurse practitioner led interdisciplinary intervention, compared with physician‐led PACT	**Proactive case finding—mixed** The imPACT patients had significantly greater referrals to hospice, suggesting better identification of palliative care needs. This was no longer significant in sensitivity analysis which removed people with cancer and dementia
Jackson et al., 2016 USA (Jackson et al., 2016)	Retrospective analytical cohort study	35,174 (7468 intervention, 27,706 control). No age restriction, multimorbid population identified using predictive modelling	91%	Nurse home visits in addition to an existing transitional care intervention, compared with transitional care intervention without home visit	**Healthcare use—mostly improved** Home visits generated strong reduction in 30‐day readmission and reduced 6‐month inpatient admission in 4 of 6 subgroups stratified by risk. **Costs—mixed** 6‐month inpatient costs were reduced in the highest risk strata but were unaffected in the remaining five subgroups
Karlsson & Karlsson, 2019 Sweden (Karlsson & Karlsson, 2019)	Qualitative (descriptive/exploratory)	10 nurses providing home visits to people aged ≥65 with multimorbidity	50%	Follow‐up 48–72 transitional care intervention. Nurse home visits post‐hospital discharge	**Quality of healthcare—mostly improved** Some illustration of nurses improving satisfaction with healthcare by answering questions/ relieving anxieties at home visit **Communication—mostly improved** Some illustration to suggest face‐to‐face visit improves ability to assess patients holistically
Lupari, 2011 UK, Northern Ireland (Lupari, 2011)	Mixed‐methods: controlled (quasi‐ experimental) clinical trial/ qualitative descriptive	590 (295 intervention, 295 control) Age > 65, multimorbid population identified using predictive modelling	Quant: 89% Qual: 90%	Community‐based nurse case management intervention	**Health‐related quality of life—mostly improved** Health‐related quality of life improved across the three timepoints (12,24,36 months) for the intervention, control declined [EQ‐5D + VAS] **Healthcare use—mostly improved** Bed days significantly reduced at all timepoints apart from 24 weeks. Hospitalization significantly reduced at 36 weeks but not 12 or 24. **Costs—mostly improved** Intervention group demonstrated a significant increase in QALYs at 9 months **Physical function—mostly improved** Function improved in the intervention group across the three time points while control declined [FIM/FAM] **Caregiver support—mixed** There was no significant difference between groups in caregiver strain [Caregiver Strain Index], however, qualitative illustrations suggested carers were supportive of intervention
Mallow et al., 2018 USA (Mallow et al., 2018)	Pre/post‐test (quasi‐experimental) study	30. Age ≥ 18, requires combination of chronic diseases and biopsychosocial risk factors (low income, uninsured, poor access to healthcare)	44%	mI SMART web application and telehealth intervention delivered by nurse practitioner	**Physiological measures—mostly improved** The mI SMART intervention was associated with significant improvements in blood pressure and glycaemic control, but only borderline significant weight reductions
Markle‐Reid et al., 2016 Canada (Markle‐Reid et al., 2016)	Pre/post‐test (quasi‐experimental) study (pilot)	45 (37 completed follow‐up) Age ≥ 65, requires diagnosis of diabetes and multimorbidity (≥2 other conditions)	78%	Aging, Community and Health Research Unit Community Partnership Program (ACHRU‐CPP) Nurse‐led interdisciplinary intervention to support self‐management	**Mental health—mostly unaffected** No significant differences in depressive symptoms [CES‐D] or anxiety [GAD‐7] **Health‐related quality of life—mixed** Small significant improvement in physical component of [SF‐12], no improvement in mental component **Costs—mixed** Small cost reductions in some areas of healthcare offset an increase in the cost of diabetes care **Self‐management behaviour—mostly unaffected** No improvements detected in diabetes self‐care [Diabetes Self Care Activity Scale]
Markle‐Reid et al., 2020 Canada (Markle‐Reid et al., 2018; Miklavcic et al., 2020)	Randomized controlled trial	2 sites: Ontario: 159 (80 intervention, 79 control) Alberta: 132 (70 intervention, 62 control) Age ≥ 65, requires diagnosis of diabetes and multimorbidity (≥2 other conditions)	77%	Aging, Community and Health Research Unit Community Partnership Program (ACHRU‐CPP) Nurse‐led interdisciplinary intervention to support self‐management.	**Mental health—mixed** Difference in depression [CES‐D‐10] favouring the intervention group in Ontario. No difference for depression in Alberta. Anxiety [GAD‐7] unaffected at both sites. **Health‐related quality of life—mixed** The mental component score of [SF‐12] was higher in the Ontario intervention group. There was no difference in mental component score in Alberta, and there was no difference in physical component score at either site. **Costs—mixed** At both sites, small reductions in costs across healthcare services were offset by an increase in diabetes‐related costs, indicating cost neutrality. **Self‐management behaviour—mixed** There was a small significant improvement in diabetes self‐management in Ontario. No difference was detected in Alberta [Summary of Diabetes Self‐Care Activities] **Self‐efficacy—mostly unaffected** No significant difference was detected in self‐efficacy at either site [Self Efficacy for Managing Chronic Disease Scale]
Moran et al., 2008 UK, Wales (Moran et al., 2008)	Pre/post‐test (quasi‐experimental) study	116. Phase1: age ≥ 65, 2 or more chronic conditions and hospitalization risk factors. Phase 2: age ≥ 50 ≥ 1 chronic condition	67%	Flintshire case‐management intervention. Community‐based case‐management intervention	**Healthcare use—mixed** 12‐month post‐intervention ED admissions were significantly lower, but differences in length of inpatient stay were not significant
Randall et al., 2015 UK, England (Randall et al., 2014, 2015)	Qualitative (descriptive/exploratory)	2 phases: Phase 1: 43 Phase 2: 45 Community matrons, patients, carers, managers, former commissioners, GPs, secondary care staff	80%	Community Matrons Community‐based advanced nurse case‐management intervention	**Quality of healthcare—mixed** There were many illustrations where patients and staff felt patient care had been improved by the intervention, but also gaps identified particularly in communicating with secondary care. **Communication—mostly improved** Communicating with the community matrons was seen as easy and preferable to going through GP surgery **Self‐management behaviour—mostly improved** There were illustrations of participants engaged in good self‐management behaviours, which were credited to the community matrons' intervention
Sadarangani et al., 2019 USA (Sadarangani et al., 2019)	Mixed methods: pre/post‐test (quasi‐experimental) study, with qualitative descriptive/exploratory interviews	Quantitative phase: 126. Age ≥ 18, combination of >1 chronic conditions, psychosocial conditions and hospitalization risk. Qualitative phase: 40 stakeholders (patients, carers, administrators, nurses, social workers)	Quant: 67% Qual: 80%	Community‐based health home. Case‐management intervention delivered by a registered nurse navigator with interdisciplinary team	**Mental health—mostly improved** Severe depression was reduced at 12 months [Geriatric Depression Scale]. There was also a reduction in loneliness [UCLA Loneliness Scale], and a borderline significant improvement in cognitive impairment [Orientation Memory Cognition Tool] **Health‐related quality of life—mostly improved** There was a significant increase in the number of people reporting good quality of life at 12 months [Revised Dementia Quality of Life Self‐Esteem subscale] **Healthcare use—mixed** There was a significant reduction in ED use over the preceding 12 months but no difference in hospitalization. **Prioritization—mostly improved** There was qualitative evidence of nurses engaging in patient‐centred goal setting, and that patients appreciated this approach **Self‐management behaviour—mostly improved** There was qualitative evidence of patients adopting positive health behaviours as a result of intervention **Nutrition—mostly improved** There was a significant reduction in proportion of those at ‘high nutritional risk’ at 12 months [DETERMINE checklist] **Falls risk—mostly unaffected** The proportion of those at ‘high‐risk’ of falls was marginally higher post‐intervention [STEADI assessment]. Authors attribute this to likely disease progression or improved detection of high‐risk individuals. **Pain—mostly improved** There was a significant reduction in the number of people reporting poorly controlled pain at 12 months [Modified Universal Pain Assessment Tool] **Proactive case‐finding—mixed** There was qualitative‐only evidence to indicate the intervention improved early detection of high‐risk patients and appropriate referral **Disease management—mostly improved** There was qualitative evidence to indicate nurses provided effective disease management and early detection of deterioration in health status **Trust and advocacy—mostly improved** There was qualitative evidence to indicate nurses provided an advocacy role for the patients with other health and social care providers **Caregiver support—mostly improved** There was qualitative evidence to suggest nurses were involved in proactively identifying and supporting caregivers
Steinman et al., 2018 Israel (Steinman et al., 2018)	Cluster‐controlled (quasi‐experimental) clinical trial	1218 (622 intervention, 596 control). Age 45–94, 3 or more chronic conditions, high hospitalization risk determined by predictive modelling	89%	Comprehensive Care of Multimorbid Adults Project (CC‐MAP) Primary care case‐management intervention, based on Guided Care model	**Prioritization ‐ mostly improved** There was a significant increase in the number of medication changes and symptom‐focussed medication changes (yet no increase in number of medications) in the intervention group, interpreted as a ‘fine‐tuning’ of medications in a patient‐centred manner
Taveira et al., 2019 Portugal (Taveira et al., 2019)	Pre/post test (quasi‐experimental) study	50. Adults (no age restriction) ≥2 chronic illnesses	33%	Primary care nurse case manager within an integrative care team	**Healthcare use—mostly improved** Reductions in ED admission, basic ED usage, family doctor and specialist consultations and inpatient admissions were all reduced.
Valdivieso et al., 2018 Spain (Valdivieso et al., 2018)	Controlled (quasi‐experimental) clinical trial (3 arms)	472 (78 telehealth arm, 168 phone arm, 170 control) Age ≥ 18, multimorbid population identified using predictive modelling	56%	Hospital case‐management nurse intervention. Phone arm made contact by telephone only, telehealth arm also used Bluetooth‐enabled devices for physiological monitoring.	**Mortality—mostly unaffected** No differences detected in mortality between groups at 12 months **Mental health—mostly improved** The telephone group saw a reduction in cognitive impairment [Pfeiffer Score] over 12 months, but not the telehealth group **Health‐related quality of life—mostly improved** Both intervention arms were associated with improved [EQ‐5D] scores, and the telehealth group also had improved [EQ‐5D VAS] at 12 months **Activities of daily living—mostly improved** The telephone group had improved functional ability/ADLs at 12 months compared with control [Barthel Index] **Healthcare use ‐ mostly unaffected** There was no reduction between groups for any aspect of healthcare utilization.

Abbreviations: ADL, activities of daily living; CM, case manager; ED, emergency department; GC, Guided Care; ICU, intensive care unit; QALY, quality‐adjusted life years; T2DM, type 2 diabetes mellitus.

^a^ Study title decided based on either the principal investigator (if known), or the first author of the most recent or significant publication. This does not signify authorship as determined by the publication authors.

^b^ Summary quality scores calculated as a percentage of the domains in the scoring tool which were scored ‘yes’.

^c^ Outcome measures in parentheses where identified by study authors.

### 4.2 Quality of included studies

The median summary quality score was 77.5% (interquartile range [IQR]: 58.8–89.0) for all studies (see Table 3). Two smaller quasi‐experimental studies scored less than 50% but did not add unique findings. The most common issues in RCTs related to blinding of participants or clinicians (*n* = 3). Absence of control groups (*n* = 5) and single measurements of outcomes (*n* = 6) were common in quasi‐experimental studies. Qualitative studies lacked statements locating the researcher culturally or theoretically (*n* = 5) and most did not consider the impact of the researcher on the research (*n* = 3). Failure to discuss theory and reflexivity in qualitative research suggests that studies have been conducted in a value‐neutral or atheoretical manner, which is at odds with the assumptions of most qualitative approaches (Braun & Clarke, 2021; Sandelowski, 1993) and limits the transferability of findings. We have highlighted which results are based purely on qualitative findings. File S2 provides further detail.

**TABLE 3:** Summary quality scores

Study type	Number of studies^a^	Summary quality score		
		Median	Range	Interquartile range
Randomized controlled trials	3	77.0	77–85	77.0–81.0
Quasi‐experimental studies	10	67.0	33–89	58.8–86.3
Retrospective cohort studies	4	73.0	55–100	55.0–93.3
Qualitative studies	5	80.0	50–90	80.0–90.0
All studies	20	77.5	33–100	58.8–89.0

^a^ Number of studies greater than 20. Mixed‐methods studies scored on both qualitative and quantitative components.

### 4.3 Participant characteristics

The average age of participants was reported in 13 studies and ranged from 38.0 to 83.1 (median: 75.6, IQR: 73.9–76.5), reflecting the fact that most (*n* = 11) studies targeted older adults. Nine studies reported an average number of chronic conditions, ranging from 2.85 to 11.5 (median: 4.45, IQR: 4.02–8.00). All studies recruited participants from both sexes and were broadly evenly distributed.

### 4.4 Identification of multimorbidity

Ten studies required two or more chronic conditions to be eligible (Chow & Wong, 2014; García‐Fernández et al., 2014; Gustafsson et al., 2013; Hjelm et al., 2015; Karlsson & Karlsson, 2019; Markle‐Reid et al., 2016, 2018; Miklavcic et al., 2020; Moran et al., 2008; Randall et al., 2014, 2015; Sadarangani et al., 2019; Taveira et al., 2019). Two of these studies—a pilot study and subsequent RCT (Markle‐Reid et al., 2016, 2018; Miklavcic et al., 2020)—also required a diagnosis of diabetes, but the intervention was multimorbidity‐focussed. Seven studies used predictive models to identify intensive healthcare users with multimorbidity (Boult et al., 2008, 2011; Boyd et al., 2010, 2008; Hanson et al., 2018; Hummel et al., 2017; Jackson et al., 2016; Leff et al., 2009; Lupari, 2011; Sylvia et al., 2008; Takahashi et al., 2016; Valdivieso et al., 2018). One study required three conditions and also employed a predictive model (Steinman et al., 2018). The remaining two studies identified multimorbid populations through clinician judgement (Dorr et al., 2008) and a combination of chronic disease and risk factors (Mallow et al., 2018).

### 4.5 Intervention components

**FIGURE 2:**
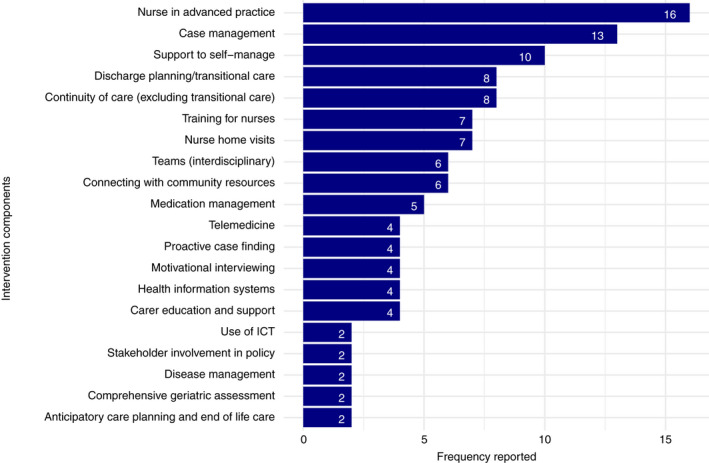
Intervention components

File S3 details the adapted EPOC taxonomy. Interventions were complex, with the average intervention comprising 5.75 components (range 2–12). Figure 2 outlines components by frequency.

Common components included nurses in advanced practice (*n* = 16), case‐management (*n* = 13) supporting self‐management (*n* = 10), discharge planning/transitional care (*n* = 8) and interventions to improve continuity of care (*n* = 8). A common theme was involvement of an experienced nurse managing complex care to reduce healthcare utilization or improve the quality of patient care. Interventions were delivered either as a continuous intervention (with the nurse serving as primary caregiver) or targeted at high‐risk transitions, such as hospital discharges.

### 4.6 Intervention type and the effect on outcomes

**FIGURE 3:**
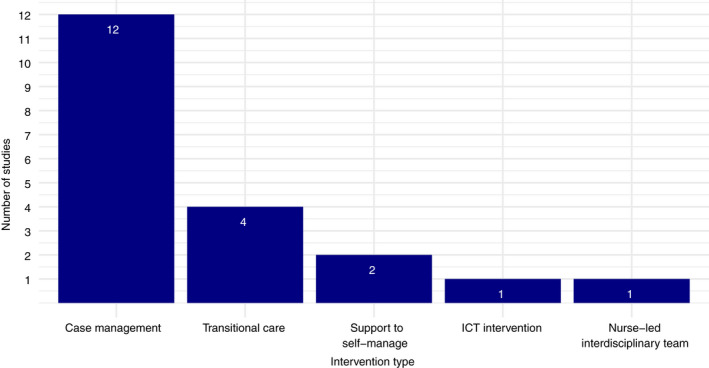
intervention types

Interventions were further categorized by the component which most accurately summarized them overall (Figure 3). Most were case‐management interventions (*n* = 12), followed by transitional care interventions (*n* = 4), interventions to support self‐management (*n* = 2), nurse‐led interdisciplinary team interventions (*n* = 1) and ICT interventions (*n* = 1).

Table 4 outlines the effects recorded in included studies, grouped by intervention type.

**TABLE 4:** The effect of interventions on outcomes

	Case‐management	Transitional care	Supported self‐management	Nurse‐led interdisciplinary team	ICT interventions
Health‐related quality of life	**Mostly improved** (Lupari, 2011; Sadarangani et al., 2019; Valdivieso et al., 2018)	**Mostly improved** (Chow & Wong, 2014)	**Mixed** (Markle‐Reid et al., 2016, Markle‐Reid et al., 2018; Miklavcic et al., 2020)		
Mental health	**Mixed** (Sadarangani et al., 2019; Valdivieso et al., 2018)		**Mostly unaffected** (Markle‐Reid et al., 2016, Markle‐Reid et al., 2018; Miklavcic et al., 2020)		
Mortality	**Mixed** (Dorr et al., 2008; García‐Fernández et al., 2014; Valdivieso et al., 2018)	**Mixed** (Takahashi et al., 2016)			
Self‐rated health		**Mostly improved** (Chow & Wong, 2014)			
Self‐management behaviour	**Mostly improved** (Randall et al., 2014, Randall et al., 2015; Sadarangani et al., 2019)^a^		**Mixed** (Markle‐Reid et al., 2016, Markle‐Reid et al., 2018; Miklavcic et al., 2020)		
Self‐efficacy		**Mostly improved** (Chow & Wong, 2014)	**Mostly unaffected** (Markle‐Reid et al., 2018; Miklavcic et al., 2020)		
*Caregiver burden/support*	**Mixed** (García‐Fernández et al., 2014; Lupari, 2011; Sadarangani et al., 2019)				
*Pain*	**Mostly improved** (Sadarangani et al., 2019)				
Activities of daily living	**Mostly improved** (García‐Fernández et al., 2014; Valdivieso et al., 2018)				
Physical function	**Mixed** (Lupari, 2011)				
*Physiological measures*					**Mixed** (Mallow et al., 2018)
*Disease management*	**Mostly improved** (Sadarangani et al., 2019)^a^				
*Nutrition*	**Mostly improved** (Sadarangani et al., 2019)				
*Falls risk*	**Mostly unaffected** (Sadarangani et al., 2019)				
Communication	**Mostly improved** (Randall et al., 2014, Randall et al., 2015)^a^	**Mixed** (Karlsson & Karlsson, 2019)^a^			
Prioritization	**Mostly improved** (Sadarangani et al., 2019; Steinman et al., 2018)				
*Trust and advocacy*	**Mostly improved** (Gustafsson et al., 2013; Hjelm et al., 2015; Sadarangani et al., 2019)^a^				
Healthcare use	**Mixed** (Boult et al., 2011; Dorr et al., 2008; García‐Fernández et al., 2014; Lupari, 2011; Moran et al., 2008; Sadarangani et al., 2019)	**Mostly improved** (Chow & Wong, 2014; Jackson et al., 2016; Takahashi et al., 2016)			
Costs	**Mixed** (Leff et al., 2009; Lupari, 2011; Sylvia et al., 2008)	**Mixed** (Hanson et al., 2018; Jackson et al., 2016)	**Mixed** (Markle‐Reid et al., 2016, Markle‐Reid et al., 2018; Miklavcic et al., 2020)		
Quality of healthcare (patient‐rated)	**Mostly improved** (Boult et al., 2008; Boyd et al., 2010, Boyd et al., 2008; García‐Fernández et al., 2014; Hjelm et al., 2015; Randall et al., 2014, Randall et al., 2015)	**Mixed** (Karlsson & Karlsson, 2019)^a^			
*Quality of healthcare (nurse/physician rated)*	**Mixed** (Boyd et al., 2010; Randall et al., 2014)				
*Case‐finding*	**Mixed** (Sadarangani et al., 2019)			**Mixed** (Hummel et al., 2017)	

*Note*: Italicized outcomes not COSmm outcomes and were coded inductively.

*Note*: Mostly Improved = green, Mixed = amber, Mostly unnafected = red.

^a^ Qualitative evidence only.

#### 4.6.1 Case‐management interventions

Case‐management interventions were characterized by comprehensive patient assessment, tailored care planning, (Boult et al., 2011; Boyd et al., 2008; Chow & Wong, 2014; Sadarangani et al., 2019; Steinman et al., 2018) and often focussed on improving continuity of care (Boult et al., 2011; Boyd et al., 2008; Lupari, 2011; Steinman et al., 2018; Taveira et al., 2019). Some case‐managers had advanced clinical skills (Lupari, 2011; Randall et al., 2014), and were the primary healthcare provider, while others worked in partnership with the patient's primary care team (Boult et al., 2011; Boyd et al., 2008; Steinman et al., 2018). Several interventions required that case‐managers undertake bespoke training (Boult et al., 2011; Boyd et al., 2008; Lupari, 2011; Moran et al., 2008; Steinman et al., 2018). Nurse case‐managers were present across primary care (Boult et al., 2011; Boyd et al., 2008; Dorr et al., 2008; Steinman et al., 2018; Taveira et al., 2019), secondary care (García‐Fernández et al., 2014; Valdivieso et al., 2018) and community settings (Hjelm et al., 2015; Lupari, 2011; Moran et al., 2008; Randall et al., 2015; Sadarangani et al., 2019).

##### Effect of case‐management interventions

Case‐management interventions had positive effects on health‐related quality of life, self‐management behaviour, pain and disease management, nutrition, activities of daily living, communication with healthcare providers, prioritization of needs, fostering trust and advocacy and the overall quality of care from the patient perspective (Table 4).

Effects on health services were mixed. While some studies noted reductions in bed‐days and emergency care use for community‐based interventions (Lupari, 2011; Sadarangani et al., 2019), two large studies of primary care interventions did not detect a reduction across the majority of interactions with healthcare services (Boult et al., 2011; Dorr et al., 2008). Two hospital‐based interventions also did not detect reductions in healthcare use (García‐Fernández et al., 2014; Valdivieso et al., 2018). Cost reduction and mortality were similarly mixed. A community intervention in Northern Ireland demonstrated a significant reduction in healthcare costs (Lupari, 2011), while a primary care‐based intervention from the United States failed to generate significant reductions (Leff et al., 2009; Sylvia et al., 2008). Two studies involving hospital case‐managers did not detect any reduction in mortality at 90 days (García‐Fernández et al., 2014) and 12 months (Valdivieso et al., 2018), while a large study of a community‐based nurse case‐manager intervention (3432 participants) found significant differences in the proportion of deaths favouring the intervention group at 1 year, although despite a crude reduction at 2 years, significance was not sustained (Dorr et al., 2008).

Mixed effects were also noted for mental health, specifically depression, loneliness and cognitive impairment (Sadarangani et al., 2019; Valdivieso et al., 2018), as well as caregiver support (García‐Fernández et al., 2014; Lupari, 2011; Sadarangani et al., 2019), physical functioning (Lupari, 2011), and quality of care from physician perspective (Boyd et al., 2010; Randall et al., 2014). Qualitative findings suggested case‐finding for referral to other services may be improved by a community‐based intervention, and quantitative findings indicated that there was no reduction in falls risk as a result of the same intervention (Sadarangani et al., 2019).

#### 4.6.2 Transitional care interventions

Transitional care interventions are relatively short‐term and focus on the coordination of patient care and changes to the care process (EPOC, 2015); the interventions included in this review all targeted the transition from acute hospital to home (Chow & Wong, 2014; Hanson et al., 2018; Jackson et al., 2016; Karlsson & Karlsson, 2019). Home visits were common to all interventions however, one study compared a combined telephone and home visit service with a telephone‐only service (and usual care) (Chow & Wong, 2014). One study examined the effect of a home visit by a nurse nested within an existing transitional care intervention (Jackson et al., 2016).

##### Effect of transitional care interventions

Transitional care interventions demonstrated positive effects on health‐related quality of life, self‐rated health, self‐efficacy and healthcare use (Table 4). One study evidenced improvements in physical functioning, self‐efficacy, self‐rated health and reduced readmission within 84 days of discharge, although not within 28 days (Chow & Wong, 2014). Conversely, a propensity‐matched cohort analysis of another intervention demonstrated a reduction in readmission and ED attendance at 30 days which was not sustained at 90 or 180 days (Takahashi et al., 2016). In a large US study (35,174 participants), the addition of a nurse home visit to an existing transitional care intervention also generated positive reductions in 30‐day readmission, as well as reduced 6‐month readmission in four of six risk strata (Jackson et al., 2016).

Mortality results were mixed, with evidence of un‐sustained reductions in one study (Takahashi et al., 2016). Cost reductions were only evidenced for those with the highest risk of healthcare utilization, as determined by predictive modelling (Hanson et al., 2018; Jackson et al., 2016), suggesting that targeting such groups in this way may be cost‐effective. Limited qualitative findings suggested a Swedish intervention may have improved communication and quality of healthcare from the patient perspective (Karlsson & Karlsson, 2019).

#### 4.6.3 Supported self‐management interventions

Two studies from the same programme of research were predominantly focused on supporting self‐management. The six‐month intervention included home visits, group education sessions, case conferences and nurse‐led care coordination. There were variations in delivery between study phases and sites, however, the above components were consistent throughout (Markle‐Reid et al., 2016; Markle‐Reid et al., 2018; Miklavcic et al., 2020).

##### Effect of supported self‐management interventions

Effects varied between reports. It did not generate an overall reduction in costs, yet savings in some areas were explained to offset increases in others, achieving cost‐neutrality. Mixed results were reported across studies for health‐related quality of life and self‐management. No improvement was noted in mental health and self‐efficacy (Markle‐Reid et al., 2016, 2018; Miklavcic et al., 2020).

#### 4.6.4 Nurse‐led interdisciplinary team interventions

Interdisciplinary team working featured in six interventions, however, only one study specifically focussed on evaluating the effect of a nurse practitioner‐led intensive management patient‐aligned care team, comprised of a nurse practitioner (team leader), social worker, recreation therapist, administration coordinator and a part‐time physician champion. This was compared with a less intensive physician‐led team. The key features of the intervention involved 24/7 contact to either the nurse practitioner or physician, comprehensive interdisciplinary assessment and frequent follow‐up, health education and coaching, hospital ‘in‐reach’, co‐attendance at appointments and connecting patients with community resources (Hummel et al., 2017).

##### Effect of nurse‐led interdisciplinary team interventions

The intervention reported a crude improvement in identification and referral of people with palliative and end‐of‐life care needs, however, when the imbalanced distribution of people with cancer and dementia was removed in a sensitivity analysis, the difference was no longer significant (Hummel et al., 2017).

#### 4.6.5 Information and communication technology (ICT) interventions

The use of ICT and telehealth was present in many interventions, however only one intervention evaluated the effect of a nurse practitioner‐led smart technology intervention to a cohort of adults with multimorbidity. The 12‐week intervention made use of a patient‐facing web application provided to participants on a tablet computer alongside a range of devices for physiological monitoring, such as Bluetooth‐enabled scales, glucometer and sphygmomanometer. Participants were expected to use the application in place of attending the clinic and the nurse acted as primary clinician (Mallow et al., 2018).

##### Effect of ICT interventions

The small (30 participants) study reported improvements in some physiological measures (blood pressure and glycaemic control) but not others (weight control; Mallow et al., 2018).

## 5 DISCUSSION

We have found that nurse‐led interventions for multimorbidity are mostly either case‐management or transitional care interventions, often employ a nurse in advanced practice and are focussed on simplifying care and supporting self‐management. Effects on outcomes were mixed; case‐management had positive effects on patient‐centred outcomes, yet the impact on health service outcomes was inconsistent. Transitional care interventions were more consistent in reducing healthcare usage and reattendance, albeit with variations in the longevity of the effect. The overall aim of this review was to identify the types of nurse‐led interventions which improve outcomes for people with multimorbidity; we conclude that interventions which focus on coordination and management of care in both the long‐ and short‐term have the potential to improve patient‐centred and patient‐reported outcomes but are less consistent in improving health‐service oriented outcomes such as usage and costs.

To better understand the types of nurse‐led interventions which are most effective, we should locate these interventions in relation to established theory. First, interventions were generally grounded in the Chronic Care Model (Wagner et al., 2002). Importance was placed on the organization of healthcare services, and on connecting nurses and patients with resources available in the wider community. Productive interactions between nurse and patient were seen as essential to the shared development of care plans and to setting realistic goals and priorities about the individuals' care. Both case management and transitional care interventions tend to operate within this framework, albeit over different durations and in different settings.

Nested within the Chronic Care Model is the concept of supported self‐management, which also underpinned many interventions in this review. Yet, studies which measured the effect interventions had on treatment burden were conspicuously absent, despite this being an important outcome to consider in multimorbidity research (Smith et al., 2018) and one which can negatively impact an individual's ability to self‐manage multimorbid conditions (Boyd et al., 2014). Burden of Treatment Theory describes the complex interplay between the individual, the health system and their social network, and how these relationships mediate the individual's capacity to self‐manage effectively (May et al., 2014). Validated tools exist to measure treatment burden in people with multimorbidity (Tran et al., 2014), and these should be deployed in future studies of similar interventions.

While interventions were spread between primary, secondary and community care, their orientation is closest to the generalist and person‐centred approach of primary care. A primary care approach to multimorbidity care should include comprehensive assessment which considers interactions between the many drugs and conditions a person has, prioritization of care based on patient preferences, and individualized management and follow‐up. All this should be centred around realistic and shared treatment goals (Muth et al., 2014).

We should also consider where this approach aligns with nursing models. At their most fundamental level, we suggest these interventions are grounded in primary nursing theory, where an individual nurse is responsible for an individual patient and for providing continuous care to them. This is in contrast with alternative and more common models such as team‐based nursing (where nurses work together to care for a group of patients) and patient‐allocation (where nurses care for a different allocation of patients each shift) (Fernandez et al., 2012). A primary nursing approach requires experienced nurses who are well‐prepared to undertake this level of practice, which we note in the large number of interventions which required a nurse undertaking various levels of advanced practice.

Despite these elements of nurse‐led interventions being present across case‐management and transitional are interventions, the effect they had on outcomes was varied. Small improvements in patient‐centred outcomes coupled with lesser impacts on healthcare services and health/disease outcomes are common features of most evaluations of multimorbidity interventions (Crowe et al., 2016; Smith et al., 2021) and this review finds that nurse‐led interventions are no different. Interventions for this patient group are complex and multi‐faceted, deployed within complex healthcare systems and target patients who are, by definition, also complex. These layers of complexity reflect the real‐world challenges of developing and testing interventions for this group. It is challenging and resource‐intensive to isolate the effects of the components or system factors which are responsible for observed effects (Poitras et al., 2018), but such approaches may be necessary in the future development of nurse‐led interventions for people with multimorbidity. Adopting a primary care and primary nursing‐based approach may provide a foundation on which to build such interventions.

### 5.1 Limitations

Identifying studies concerning multimorbidity is challenging due to inconsistent terminology (Nicholson et al., 2019). We designed our definition of multimorbidity to account for this, however, it is possible some studies may have been missed if not identified by their authors as being concerned with multimorbidity. A similar problem exists in defining nurse‐led interventions, although we sought to ensure the studies included met our agreed definition. Finally, only articles published in English were included, therefore some non‐English studies may have been missed.

We adopted a convergent‐integrated design primarily because we identified significant heterogeneity in study design and intervention type through scoping and because we wanted to include qualitative evidence in our analysis. Measuring certainty (through systems such as GRADE) is not recommended in convergent‐integrated reviews, therefore we have been cautious in our interpretation of intervention effects. A similar issue exists regarding quality appraisal; we have applied a summary quality score to provide a summary of the extent to which quality outcomes were satisfied, but it is important to note that this does not allow for comparisons to be made between studies with different designs. Full details of quality assessment can be found in File S2.

### 5.2 Future research

For those developing and evaluating complex interventions for people with multimorbidity, future research should focus on evaluating the components within these interventions, to better distil which elements are effective. RCTs can be conducted within existing interventions, and qualitative methods may be able to guide the selection of intervention components to be analysed. The way we interact with healthcare services has also been impacted by the COVID‐19 pandemic, and while some countries are moving towards a degree of ‘normality’ in their practices, the effect of remote consultations, self‐testing, shielding and such may have altered perspectives on what is desirable in an intervention. Further research should explore this in the context of multimorbidity.

Treatment burden is an important factor in multimorbidity research, and future evaluations should make use of validated tools to measure the effect of interventions on this outcome. Finally, researchers should strive to ensure a uniform terminology is employed to define multimorbidity in studies of interventions.

### 5.3 Implications for practice

Nurses are well‐placed to manage people with multimorbidity, as the transdiagnostic approach required compliments the person‐centred approach of nursing care. Nurses working with people with multimorbidity should encourage involvement in decisions about their care and endeavour to create and regularly review care plans which align with the person and their needs.

## 6 CONCLUSION

Nurse‐led interventions for multimorbidity are characterized by the development and review of individualized patient‐centred care plans by a primary nurse responsible for continuous care, working in partnership with the patient. They may constitute long‐term interventions such as case management, or they may be brief and targeted at high‐risk periods (such as transitional care). The evidence indicates that both approaches are acceptable to patients and may increase satisfaction, but the evidence for health service improvements is less clear. As researchers and practitioners, we need to consider the complexity of such interventions and the patients for whom they are designed, and we must plan our evaluations and care accordingly.

## AUTHORS' CONTRIBUTIONS

All authors have agreed on the final version and meet at least one of the following criteria (recommended by the ICMJE*):

1. substantial contributions to conception and design, acquisition of data, or analysis and interpretation of data;
2. drafting the article or revising it critically for important intellectual content.

*http://www.icmje.org/recommendations/

CM, BJ and MC conceived and designed the review. CM collected data and conducted analysis with support and verification from BJ and MC. CM drafted the article. CM, BJ and MC performed critical revision of the article and approved the final version for publication.

## FUNDING INFORMATION

This study was funded by NHS Greater Glasgow and Clyde as part of the first author's PhD fellowship.

## CONFLICT OF INTEREST

No conflict of interest has been declared by the authors.

### PEER REVIEW

The peer review history for this article is available at https://publons.com/publon/10.1111/jan.15427.

## PATIENT AND PUBLIC INVOLVEMENT

A patient advisor has reviewed, approved and provided comments on this manuscript.

## Supporting information


File S1

File S2

File S3

Data S1

Data S2

## Data Availability

Data sharing is not applicable to this article as no new data were created or analysed in this study.
