# Supplementary material for: A mixed‐methods systematic review of nurse‐led interventions for people with multimorbidity
Source: J Adv Nurs. 2022 Sep 5;78(12):3930–51. doi: 10.1111/jan.15427 (PMC9826481; doi:10.1111/jan.15427)
Supplement: Supplementary file 1 — File S1 [file JAN-78-3930-s004.docx]

# Supplement 1: Database search strategies

## CINAHL

| # | Query |
| --- | --- |
| S17 | S15 AND S16 |
| S16 | S3 OR S4 OR S5 OR S6 OR S7 OR S8 OR S13 OR S14 |
| S15 | S1 OR S2 |
| S14 | TI ( (“nurse led” OR “nurse-led” OR “nurse managed” OR “nurse-managed” OR “nurse based intervention” OR “nurse-based intervention” OR “primary nurse” OR “primary nurses” OR “primary nursing” OR “nurse practitioner” OR “nurse practitioners” OR “practitioner nurse” OR “practitioner nurses” OR “advanced practice nurse” OR “advanced practice nursing” OR “advanced practice nurses” OR “nurse specialist” OR “nurse specialists” OR “specialist nurse” OR “specialist nurses” OR “specialist nursing” OR “nurse clinician” OR “nurse clinicians” OR “nurse consultant” OR “nurse consultants” OR “consultant nurse” OR “consultant nurses” OR ((“case manager” OR “case-manager” OR “case management” OR “case-management”) AND (nurse OR nurses OR nursing))) ) OR AB ( (“nurse led” OR “nurse-led” OR “nurse managed” OR “nurse-managed” OR “nurse based intervention” OR “nurse-based intervention” OR “primary nurse” OR “primary nurses” OR “primary nursing” OR “nurse practitioner” OR “nurse practitioners” OR “practitioner nurse” OR “practitioner nurses” OR “advanced practice nurse” OR “advanced practice nursing” OR “advanced practice nurses” OR “nurse specialist” OR “nurse specialists” OR “specialist nurse” OR “specialist nurses” OR “specialist nursing” OR “nurse clinician” OR “nurse clinicians” OR “nurse consultant” OR “nurse consultants” OR “consultant nurse” OR “consultant nurses” OR ((“case manager” OR “case-manager” OR “case management” OR “case-management”) AND (nurse OR nurses OR nursing))) ) |
| S13 | S9 AND S12 |
| S12 | S10 OR S11 |
| S11 | (MH "Nursing Role") |
| S10 | (MH "Nurses+") |
| S9 | (MH "Case Management") OR (MH "Case Managers") |
| S8 | (MH "Advanced Nursing Practice") |
| S7 | (MH "Primary Nursing") |
| S6 | (MH "Nursing Leaders") OR (MH "Nurse Consultants") |
| S5 | (MH "Clinical Nurse Specialists+") |
| S4 | (MH "Nurse-Managed Centers+") |
| S3 | (MH "Nurse Practitioners+") |
| S2 | (MH "Comorbidity+") |
| S1 | TI ( multimorbidity or "multi-morbidity" or "multi morbidity" or multimorbidities or "multi-morbidities" or "multi morbidities" or multimorbid or "multi-morbid" or "multi morbid" or comorbidity or "co-morbidity" or "co morbidity" or comorbidities or "co-morbidities" or "co morbidities" or comorbid or "co-morbid" or "co morbid" or "multiple chronic conditions" or "multiple chronic illnesses" or "multiple chronic diseases" or "multiple conditions" or "multiple illnesses" or "multiple diseases" or "multiple diagnoses" or "morbidity pattern" or "morbidity patterns" or polymorbidity or "poly-morbidity" or "poly morbidity" or polymorbidities or "poly-morbidities" or "poly morbidities" or polypathology or "poly-pathology" or "poly pathology" or polypathologies or "poly-pathologies" or "poly pathologies" or pluripathology or "pluri-pathology" or "pluri pathology" or multipathology or "multi-pathology" or "multi pathology" or multipathologies or "multi-pathologies" or "multi pathologies" or "multiple pathologies" or "disease cluster" or "disease clusters" ) OR AB ( multimorbidity or "multi-morbidity" or "multi morbidity" or multimorbidities or "multi-morbidities" or "multi morbidities" or multimorbid or "multi-morbid" or "multi morbid" or comorbidity or "co-morbidity" or "co morbidity" or comorbidities or "co-morbidities" or "co morbidities" or comorbid or "co-morbid" or "co morbid" or "multiple chronic conditions" or "multiple chronic illnesses" or "multiple chronic diseases" or "multiple conditions" or "multiple illnesses" or "multiple diseases" or "multiple diagnoses" or "morbidity pattern" or "morbidity patterns" or polymorbidity or "poly-morbidity" or "poly morbidity" or polymorbidities or "poly-morbidities" or "poly morbidities" or polypathology or "poly-pathology" or "poly pathology" or polypathologies or "poly-pathologies" or "poly pathologies" or pluripathology or "pluri-pathology" or "pluri pathology" or multipathology or "multi-pathology" or "multi pathology" or multipathologies or "multi-pathologies" or "multi pathologies" or "multiple pathologies" or "disease cluster" or "disease clusters" ) |

## MEDLINE

| # | Searches |
| --- | --- |
| 1 | multiple chronic conditions/ |
| 2 | exp comorbidity/ |
| 3 | (multimorbidity or "multi-morbidity" or "multi morbidity" or multimorbidities or "multi-morbidities" or "multi morbidities" or multimorbid or "multi-morbid" or "multi morbid" or comorbidity or "co-morbidity" or "co morbidity" or comorbidities or "co-morbidities" or "co morbidities" or comorbid or "co-morbid" or "co morbid" or "multiple chronic conditions" or "multiple chronic illnesses" or "multiple chronic diseases" or "multiple conditions" or "multiple illnesses" or "multiple diseases" or "multiple diagnoses" or "morbidity pattern" or "morbidity patterns" or polymorbidity or "poly-morbidity" or "poly morbidity" or polymorbidities or "poly-morbidities" or "poly morbidities" or polypathology or "poly-pathology" or "poly pathology" or polypathologies or "poly-pathologies" or "poly pathologies" or pluripathology or "pluri-pathology" or "pluri pathology" or multipathology or "multi-pathology" or "multi pathology" or multipathologies or "multi-pathologies" or "multi pathologies" or "multiple pathologies" or "disease cluster" or "disease clusters").ab,ti. |
| 4 | Primary Nursing/ |
| 5 | Practice Patterns, Nurses'/ |
| 6 | exp Nurse Practitioners/ |
| 7 | nurse specialists/ or nurse clinicians/ |
| 8 | Advanced Practice Nursing/ |
| 9 | exp Nurses/ |
| 10 | exp Nursing/ |
| 11 | exp Nurse's Role/ |
| 12 | exp Nursing Care/ |
| 13 | exp Case Management/ or exp Case Managers/ |
| 14 | 9 or 10 or 11 or 12 |
| 15 | 13 and 14 |
| 16 | ("nurse led" or "nurse-led" or "nurse managed" or "nurse-managed" or "nurse based intervention" or "nurse-based intervention" or "primary nurse" or "primary nurses" or "primary nursing" or "nurse practitioner" or "nurse practitioners" or "practitioner nurse" or "practitioner nurses" or "advanced practice nurse" or "advanced practice nursing" or "advanced practice nurses" or "nurse specialist" or "nurse specialists" or "specialist nurse" or "specialist nurses" or "specialist nursing" or "nurse clinician" or "nurse clinicians" or "nurse consultant" or "nurse consultants" or "consultant nurse" or "consultant nurses" or (("case manager" or "case-manager" or "case management" or "case-management") and (nurse or nurses or nursing))).ab,ti. |
| 17 | 1 or 2 or 3 |
| 18 | 4 or 5 or 6 or 7 or 8 or 15 or 16 |
| 19 | 17 and 18 |

## Embase

| # | Searches |
| --- | --- |
| 1 | multiple chronic conditions/ |
| 2 | exp comorbidity/ |
| 3 | (multimorbidity or "multi-morbidity" or "multi morbidity" or multimorbidities or "multi-morbidities" or "multi morbidities" or multimorbid or "multi-morbid" or "multi morbid" or comorbidity or "co-morbidity" or "co morbidity" or comorbidities or "co-morbidities" or "co morbidities" or comorbid or "co-morbid" or "co morbid" or "multiple chronic conditions" or "multiple chronic illnesses" or "multiple chronic diseases" or "multiple conditions" or "multiple illnesses" or "multiple diseases" or "multiple diagnoses" or "morbidity pattern" or "morbidity patterns" or polymorbidity or "poly-morbidity" or "poly morbidity" or polymorbidities or "poly-morbidities" or "poly morbidities" or polypathology or "poly-pathology" or "poly pathology" or polypathologies or "poly-pathologies" or "poly pathologies" or pluripathology or "pluri-pathology" or "pluri pathology" or multipathology or "multi-pathology" or "multi pathology" or multipathologies or "multi-pathologies" or "multi pathologies" or "multiple pathologies" or "disease cluster" or "disease clusters").ab,ti. |
| 4 | primary nursing/ |
| 5 | nursing practice/ |
| 6 | exp nurse practitioner/ |
| 7 | nurse specialist/ or clinical nurse specialist/ |
| 8 | advanced practice nursing/ |
| 9 | nurse consultant/ |
| 10 | case management/ or case manager/ |
| 11 | exp nurse/ |
| 12 | exp nursing/ |
| 13 | exp nursing care/ |
| 14 | 11 or 12 or 13 |
| 15 | 10 and 14 |
| 16 | ("nurse led" or "nurse-led" or "nurse managed" or "nurse-managed" or "nurse based intervention" or "nurse-based intervention" or "primary nurse" or "primary nurses" or "primary nursing" or "nurse practitioner" or "nurse practitioners" or "practitioner nurse" or "practitioner nurses" or "advanced practice nurse" or "advanced practice nursing" or "advanced practice nurses" or "nurse specialist" or "nurse specialists" or "specialist nurse" or "specialist nurses" or "specialist nursing" or "nurse clinician" or "nurse clinicians" or "nurse consultant" or "nurse consultants" or "consultant nurse" or "consultant nurses" or (("case manager" or "case-manager" or "case management" or "case-management") and (nurse or nurses or nursing))).ab,ti. |
| 17 | 1 or 2 or 3 |
| 18 | 4 or 5 or 6 or 7 or 8 or 9 or 15 or 16 |
| 19 | 17 and 18 |

## Cochrane Library (CENTRAL)

ID Search

#1 MeSH descriptor: [Multiple Chronic Conditions] this term only

#2 MeSH descriptor: [Comorbidity] explode all trees

#3 (multimorbidity or "multi-morbidity" or "multi morbidity" or multimorbidities or "multi-morbidities" or "multi morbidities" or multimorbid or "multi-morbid" or "multi morbid" or comorbidity or "co-morbidity" or "co morbidity" or comorbidities or "co-morbidities" or "co morbidities" or comorbid or "co-morbid" or "co morbid" or "multiple chronic conditions" or "multiple chronic illnesses" or "multiple chronic diseases" or "multiple conditions" or "multiple illnesses" or "multiple diseases" or "multiple diagnoses" or "morbidity pattern" or "morbidity patterns" or polymorbidity or "poly-morbidity" or "poly morbidity" or polymorbidities or "poly-morbidities" or "poly morbidities" or polypathology or "poly-pathology" or "poly pathology" or polypathologies or "poly-pathologies" or "poly pathologies" or pluripathology or "pluri-pathology" or "pluri pathology" or multipathology or "multi-pathology" or "multi pathology" or multipathologies or "multi-pathologies" or "multi pathologies" or "multiple pathologies" or "disease cluster" or "disease clusters"):ti OR (multimorbidity or "multi-morbidity" or "multi morbidity" or multimorbidities or "multi-morbidities" or "multi morbidities" or multimorbid or "multi-morbid" or "multi morbid" or comorbidity or "co-morbidity" or "co morbidity" or comorbidities or "co-morbidities" or "co morbidities" or comorbid or "co-morbid" or "co morbid" or "multiple chronic conditions" or "multiple chronic illnesses" or "multiple chronic diseases" or "multiple conditions" or "multiple illnesses" or "multiple diseases" or "multiple diagnoses" or "morbidity pattern" or "morbidity patterns" or polymorbidity or "poly-morbidity" or "poly morbidity" or polymorbidities or "poly-morbidities" or "poly morbidities" or polypathology or "poly-pathology" or "poly pathology" or polypathologies or "poly-pathologies" or "poly pathologies" or pluripathology or "pluri-pathology" or "pluri pathology" or multipathology or "multi-pathology" or "multi pathology" or multipathologies or "multi-pathologies" or "multi pathologies" or "multiple pathologies" or "disease cluster" or "disease clusters"):ab

#4 MeSH descriptor: [Primary Nursing] this term only

#5 MeSH descriptor: [Practice Patterns, Nurses'] this term only

#6 MeSH descriptor: [Nurse Practitioners] explode all trees

#7 MeSH descriptor: [Nurse Specialists] this term only

#8 MeSH descriptor: [Nurse Clinicians] this term only

#9 MeSH descriptor: [Advanced Practice Nursing] this term only

#10 MeSH descriptor: [Nurses] explode all trees

#11 MeSH descriptor: [Nursing] explode all trees

#12 MeSH descriptor: [Nurse's Role] explode all trees

#13 MeSH descriptor: [Nursing Care] explode all trees

#14 MeSH descriptor: [Case Management] explode all trees

#15 MeSH descriptor: [Case Managers] explode all trees

#16 #10 OR #11 OR #12 OR #13

#17 #14 OR #15

#18 #16 AND #17

#19 ("nurse led" or "nurse-led" or "nurse managed" or "nurse-managed" or "nurse based intervention" or "nurse-based intervention" or "primary nurse" or "primary nurses" or "primary nursing" or "nurse practitioner" or "nurse practitioners" or "practitioner nurse" or "practitioner nurses" or "advanced practice nurse" or "advanced practice nursing" or "advanced practice nurses" or "nurse specialist" or "nurse specialists" or "specialist nurse" or "specialist nurses" or "specialist nursing" or "nurse clinician" or "nurse clinicians" or "nurse consultant" or "nurse consultants" or "consultant nurse" or "consultant nurses" or (("case manager" or "case-manager" or "case management" or "case-management") and (nurse or nurses or nursing))):ti OR ("nurse led" or "nurse-led" or "nurse managed" or "nurse-managed" or "nurse based intervention" or "nurse-based intervention" or "primary nurse" or "primary nurses" or "primary nursing" or "nurse practitioner" or "nurse practitioners" or "practitioner nurse" or "practitioner nurses" or "advanced practice nurse" or "advanced practice nursing" or "advanced practice nurses" or "nurse specialist" or "nurse specialists" or "specialist nurse" or "specialist nurses" or "specialist nursing" or "nurse clinician" or "nurse clinicians" or "nurse consultant" or "nurse consultants" or "consultant nurse" or "consultant nurses" or (("case manager" or "case-manager" or "case management" or "case-management") and (nurse or nurses or nursing))):ab

#20 #1 OR #2 OR #3

#21 #4 OR #5 OR #6 OR #7 OR #8 OR #9 OR #18 OR #19

#22 #20 AND #21 in Trials

## Opengrey

(multimorbidity OR "multi-morbidity" OR "multi morbidity" OR multimorbidities OR "multi-morbidities" OR "multi morbidities" OR multimorbid OR "multi-morbid" OR "multi morbid" OR comorbidity OR "co-morbidity" OR "co morbidity" OR comorbidities OR "co-morbidities" OR "co morbidities" OR comorbid OR "co-morbid" OR "co morbid" OR "multiple chronic conditions" OR "multiple chronic illnesses" OR "multiple chronic diseases" OR "multiple conditions" OR "multiple illnesses" OR "multiple diseases" OR "multiple diagnoses" OR "morbidity pattern" OR "morbidity patterns" OR polymorbidity OR "poly-morbidity" OR "poly morbidity" OR polymorbidities OR "poly-morbidities" OR "poly morbidities" OR polypathology OR "poly-pathology" OR "poly pathology" OR polypathologies OR "poly-pathologies" OR "poly pathologies" OR pluripathology OR "pluri-pathology" OR "pluri pathology" OR multipathology OR "multi-pathology" OR "multi pathology" OR multipathologies OR "multi-pathologies" OR "multi pathologies" OR "multiple pathologies" OR "disease cluster" OR "disease clusters") AND (“nurse led” OR “nurse-led” OR “nurse managed” OR “nurse-managed” OR “nurse based intervention” OR “nurse-based intervention” OR “primary nurse” OR “primary nurses” OR “primary nursing” OR “nurse practitioner” OR “nurse practitioners” OR “practitioner nurse” OR “practitioner nurses” OR “advanced practice nurse” OR “advanced practice nursing” OR “advanced practice nurses” OR “nurse specialist” OR “nurse specialists” OR “specialist nurse” OR “specialist nurses” OR “specialist nursing” OR “nurse clinician” OR “nurse clinicians” OR “nurse consultant” OR “nurse consultants” OR “consultant nurse” OR “consultant nurses” OR ((“case manager” OR “case-manager” OR “case management” OR “case-management”) AND (nurse OR nurses OR nursing))
