# Supplementary material for: A mixed‐methods systematic review of nurse‐led interventions for people with multimorbidity
Source: J Adv Nurs. 2022 Sep 5;78(12):3930–51. doi: 10.1111/jan.15427 (PMC9826481; doi:10.1111/jan.15427)
Supplement: Supplementary file 2 — File S2 [file JAN-78-3930-s005.docx]

# Supplement 2: Quality appraisal scores

All summary scores were calculated as the percentage of questions which were marked ‘Yes’. Any rows marked ‘No’, ‘NA’ or ‘Unclear’ did not count towards the score. Summary scores do not represent any generalisable level of quality and are only intended for comparisons between studies.

## Randomised controlled trials

|  | Boult et al 2011 | Chow & Wong 2014 | Markle-Reid et al 2020 |
| --- | --- | --- | --- |
| True randomisation? | Yes | Yes | Yes |
| Allocation concealment? | Yes | Yes | Yes |
| Groups similar at baseline? | No | Unclear | Yes |
| Participants blinded? | NA | Yes | NA |
| Treating clinician blinded? | NA | NA | NA |
| Outcome assessors blinded? | Yes | Yes | Yes |
| Treatment identical apart from intervention? | Yes | Yes | No |
| Follow-up complete? | Yes | Yes | Yes |
| Analysed in randomly allocated groups? | Yes | Yes | Yes |
| Outcomes measured the same between groups? | Yes | Yes | Yes |
| Outcomes measured reliably? | Yes | Yes | Yes |
| Appropriate statistical analysis? | Yes | Yes | Yes |
| Appropriate design or deviations (e.g. cluster-RCTs)? | Yes | Yes | Yes |
| **Summary score** | 77% | 85% | 77% |

## Quasi-experimental studies

|  | Boult et al 2008 | Dorr et al 2008 | Lupari 2011 | Mallow et al 2018 | Markle-Reid et al 2016 | Moran et al 2008 | Sadarangani et al 2019 | Steinman et al 2018 | Taveira et al 2019 | Valdivieso et al |
| --- | --- | --- | --- | --- | --- | --- | --- | --- | --- | --- |
| ‘Cause’ and ‘effect’ clearly differentiated? | Yes | Yes | Yes | Yes | Yes | Yes | Yes | Yes | Yes | Yes |
| Groups similar at baseline? | No | Yes | No | Yes | Yes | Yes | Yes | No | Yes | No |
| Treatment similar apart from intervention? | Yes | Yes | Yes | Yes | Yes | Yes | Yes | Yes | Unclear | Yes |
| Was there a control group? | Yes | Yes | Yes | No | No | No | No | Yes | No | Yes |
| Multiple measures of outcome pre/post? | No | Yes | Yes | Yes | No | No | No | Yes | Unclear | Unclear |
| Follow-up complete? | No | Yes | Yes | No | Yes | Yes | Yes | Yes | No | No |
| Outcomes measured same way? | Yes | Unclear | Yes | No | Yes | Yes | Yes | Yes | Yes | Yes |
| Outcomes measured reliably? | Yes | Yes | Yes | No | Yes | Yes | No | Yes | Unclear | Yes |
| Appropriate statistical analysis? | Yes | Yes | Yes | No | Yes | No | Yes | Yes | No | No |
| **Summary score** | 67% | 89% | 89% | 44% | 78% | 67% | 67% | 89% | 33% | 56% |

## Cohort studies

|  | Garcia-Fernandez et al 2014 | Hanson et al 2018 | Hummel et al 2017 | Jackson et al 2017 |
| --- | --- | --- | --- | --- |
| Groups similar and from same population? | Yes | Yes | No | No |
| Exposures measured similarly to assign to groups? | Yes | Yes | Yes | Yes |
| Exposure measurement valid and reliable? | Yes | Yes | Yes | Yes |
| Confounding factors identified? | No | Yes | Yes | Yes |
| Strategies for confounders stated? | No | Yes | Yes | Yes |
| Free of outcome at start of study? | Yes | Yes | Yes | Yes |
| Outcome measurement valid and reliable? | Unclear | Yes | No | Yes |
| Long enough follow-up time? | Yes | Yes | Yes | Yes |
| Follow-up complete? | Unclear | Yes | No | Yes |
| Strategies to address incomplete follow-up? | Unclear | Yes | No | Yes |
| Appropriate statistical analysis? | Yes | Yes | No | Yes |
| **Summary score** | 55% | 100% | 55% | 91% |

## Qualitative studies

|  | Hjelm et al 2015 | Karlsson & Karlsson  2019 | Lupari 2011 | Randall et al 2015 | Sadarangani et al 2019 |
| --- | --- | --- | --- | --- | --- |
| Congruity between philosophical perspective and methodology? | Yes | No | Yes | Yes | Yes |
| Congruity between methodology and research question/objectives? | Yes | Yes | Yes | Yes | Yes |
| Congruity between methodology and data collection? | Yes | Yes | Yes | Yes | Yes |
| Congruity between methodology and data representation/analysis? | Yes | Yes | Yes | Yes | Yes |
| Congruity between methodology and interpretation of results? | Yes | Yes | Yes | Yes | Yes |
| Statement to locate researcher culturally or theoretically? | No | No | No | No | No |
| Influence between researcher and research addressed? | Yes | No | Yes | No | No |
| Are participants and their voices adequately represented? | Yes | No | Yes | Yes | Yes |
| Is research ethical or is there evidence of ethical approval? | Yes | Yes | Yes | Yes | Yes |
| Do the conclusions flow from the analysis/interpretation of data? | Yes | No | Yes | Yes | Yes |
| **Summary score** | 90% | 50% | 90% | 80% | 80% |

#### Appraisal tool citations

**Randomised controlled trials and quasi-experimental studies**

Tufanaru, C., Munn, Z., Aromataris, E., Campbell, J., & Hopp, L. (2020). Chapter 3: Systematic reviews of effectiveness. In E. Aromataris & Z. Munn (Eds.), *JBI Manual for Evidence Synthesis* (pp. 71-102). JBI. <https://doi.org/https://doi.org/10.46658/JBIMES-20-01>

**Cohort studies**

Moola, S., Munn, Z., Tufanaru, C., Aromataris, E., Sears, K., Sfetcu, R., Currie, M., Qureshi, R., Mattis, P., Lisy, K., & Mu, P.-F. (2020). Chapter 7: Systematic reviews of etiology and risk. In E. Aromataris & Z. Munn (Eds.), *JBI Manual for Evidence Synthesis* (pp. 217-269). JBI. <https://doi.org/https://doi.org/10.46658/JBIMES-20-01>

**Qualitative studies**

Lockwood, C., Munn, Z., & Porritt, K. (2015, Sep). Qualitative research synthesis: methodological guidance for systematic reviewers utilizing meta-aggregation. *Int J Evid Based Healthc*, 13(3), 179-187. <https://doi.org/10.1097/xeb.0000000000000062>
