# Supplementary material for: A mixed‐methods systematic review of nurse‐led interventions for people with multimorbidity
Source: J Adv Nurs. 2022 Sep 5;78(12):3930–51. doi: 10.1111/jan.15427 (PMC9826481; doi:10.1111/jan.15427)
Supplement: Supplementary file 3 — File S3 [file JAN-78-3930-s003.docx]

# Supplement 3: Adaptions to EPOC taxonomy

Minor changes were made to some subcategory names of the EPOC taxonomy to better reflect the intervention components which were being identified:

1. ‘Discharge planning’ was changed to ‘discharge planning/transitional care’, to reflect the prevalence of interventions which aimed to improve continuity of care at the point of discharge from hospital.
2. ‘Continuity of care’ was changed to ‘continuity of care (excluding transitional care)’ to differentiate between interventions which sought to improve continuity in general with those specifically targeted at hospital discharges.
3. ‘Self-management’ was changed to ‘support to self-manage’, to better reflect the role of the nurse and interdisciplinary team in supporting patients, rather than simply transferring responsibility to the patient
4. ‘Site of service delivery’ was change to ‘nurse home visits’ to improve the specificity of this subcategory
5. ‘Role expansion or task shifting’ was changed to ‘nurse in advanced practice’. This subcategory was intended to capture any intervention which was led by a nurse with and advanced role. Advanced in this instance includes any role other than ‘registered nurse’ or those related to specialty such as ‘community nurse’, ‘diabetes nurse’.

Inductive coding allowed for the identification of other components which did not fit within the EPOC Taxonomy. Additional subcategories were created and grouped under an ‘other components’ category:

1. Training for nurses – any described programme of education, including recognised academic qualifications (i.e. Masters degree) and role-specific training programmes
2. Carer education and support – any education and support provided directly to caregivers of people participating in the intervention
3. Motivational interviewing – requires that the nurse is trained in and employs motivational interviewing techniques to support changes in behaviour
4. Connecting with community resources – any instances of signposting or connecting participants with community and social care resources they may be eligible for
5. Proactive case finding – can relate to proactively identifying patients who would benefit from the intervention under investigation, or proactively identifying patients within current caseload who would benefit from referral to another service
6. Medication management – specific component of intervention intended to review medications, generally focussing on polypharmacy
7. Anticipatory care planning and end of life care – identifying and documenting treatment preferences at the end of life, or providing care at the end of life

A thematic framework was created in Nvivo 12 based on this adapted taxonomy. Data extraction forms were indexed systematically using the thematic framework (below):

| 1.1 Changes to where care is provided |
| --- |
| 1.1.1 Nurse home visits |
| 1.2 Changes to who provides care |
| 1.2.1 Nurse in advanced practice |
| 1.2.2 Support to self-manage |
| 1.3 Coordination of care and management of care process |
| 1.3.1 Case management |
| 1.3.2 Comprehensive geriatric assessment |
| 1.3.3 Continuity of care (excluding transitional care) |
| 1.3.4 Discharge planning/transitional care |
| 1.3.5 Disease management |
| 1.3.6 Teams (interdisciplinary) |
| 1.4 Information and communications technology (ICT) |
| 1.4.1 Health information systems |
| 1.4.2 Use of ICT |
| 1.4.3 Telemedicine |
| 1.5 Governance- authority and accountability |
| 1.5.1 Stakeholder involvement in policy |
| 1.5.2 Prescribing |
| 1.6 Other components |
| 1.6.1 Training for nurses |
| 1.6.2 Carer education and support |
| 1.6.3 Motivational interviewing |
| 1.6.4 Connecting with community resources |
| 1.6.5 Proactive case finding |
| 1.6.6 Medication management |
| 1.6.7 Anticipatory care planning and end of life care |
